# Early life stress induced sex-specific changes in behavior is paralleled by altered locus coeruleus physiology in BALB/cJ mice

**DOI:** 10.1016/j.ynstr.2024.100674

**Published:** 2024-09-18

**Authors:** Savannah Brannan, Lauren Garbe, Ben D. Richardson

**Affiliations:** Department of Pharmacology, Southern Illinois University – School of Medicine, Springfield, IL, 62702, USA

## Abstract

Adverse childhood experiences have been associated with many neurodevelopmental and affective disorders including attention deficit hyperactivity disorder and generalized anxiety disorder, with more exposures increasing negative risk. Sex and genetic background are biological variables involved in adverse psychiatric outcomes due to early life trauma. Females in general have an increased prevalence of stress-related psychopathologies beginning after adolescence, indicative of adolescence being a female-specific sensitive period. To understand the underlying neuronal mechanisms potentially responsible for this relationship between genetic background, sex, stress/trauma, and cognitive/affective behaviors, we assessed behavioral and neuronal changes in a novel animal model of early life stress exposure. Male and female BALB/cJ mice that express elevated basal anxiety-like behaviors and differences in monoamine signaling-associated genes, were exposed to an early life variable stress protocol that combined deprivation in early life with unpredictability in adolescence. Stress exposure produced hyperlocomotion and attention deficits (5-choice serial reaction time task) in male and female mice along with female-specific increased anxiety-like behavior. These behavioral changes were paralleled by reduced excitability of locus coeruleus (LC) neurons, due to resting membrane potential hyperpolarization in males and a female-specific increase in action potential delay time. These data describe a novel interaction between sex, genetic background, and early life stress that results in behavioral changes in clinically relevant domains and potential underlying mechanistic lasting changes in physiological properties of neurons in the LC.

## Highlights

• BALB/cJ mice exposed to early life stress are hyperactive and display attention deficits.
• Only female BALB/cJ mice develop increased anxiety-like behavior after early life stress.
• Early life stress causes reduced excitability of locus coeruleus neurons in BALB/cJ mice.
• Locus coeruleus neurons from female BALB/cJ mice exposed to early life stress displayed an increase in action potential delay time.

## 1 Introduction

Adverse childhood experiences (ACEs) are associated with psychiatric disorder risk, with an estimated 44.6% of childhood onset and ∼30% of late onset psychiatric disorders being due to childhood adversity (Alkema et al., 2024; Green et al., 2010; McKay et al., 2022; McLaughlin et al., 2012). Complex interactions between several factors, including genetic background, sex, and ACE type(s) affect susceptibility to a range of psychiatric disorders, including attention deficit hyperactivity disorder (ADHD), anxiety, and depression (Golm et al., 2020, 2021; Rosen et al., 2019; Tibu et al., 2016; Wade et al., 2022). Not only is disruption of monoamine signaling-associated genes implicated in cognitive and affective disorder susceptibility, but biological sex and ACEs interact to affect related outcomes (Baumann et al., 2013; Castro Gonçalves et al., 2022; DeYoung et al., 2011; Enoch et al., 2003; Tadic et al., 2003; Voltas et al., 2015). Unfortunately, how genetic predisposition, critically-timed stress, and sex influence susceptibility to developing cognitive/affective processing deficits is unclear.

Based on documented sex-dependent stress-induced changes in activity of noradrenergic neurons in the locus coeruleus (LC) (Bangasser et al., 2010, 2013; Borodovitsyna et al., 2022; Curtis et al., 2006; Fan et al., 2014; Jacobson, 2019; Keshavarzy et al., 2015; Nakamoto et al., 2017; Nishinaka et al., 2015; Sheppard et al., 2024) and the role of the LC in diverse cognitive and affective processes (Campese et al., 2017; Carlson et al., 2021; Lim et al., 2010; Mair et al., 2005; McCall et al., 2017a; Uematsu et al., 2017), we hypothesize that critically-timed stress exposure may alter processing in the LC to shape multiple behaviors. Norepinephrine (NE) released from LC neurons widely projecting throughout the central nervous system is a well-established major modulator of arousal. Decades of work in animal models indicate that LC neuron projections to the medial prefrontal cortex (mPFC) are involved in maintaining attention and arousal (Ishida et al., 2000; Lim et al., 2010; Mair et al., 2005; Pasquier and Reinoso-Suarez, 1978). In parallel, LC neurons projecting to the basolateral and central amygdala (BLA and CeA) are involved in anxiety and emotional learning behaviors (Campese et al., 2017; McCall et al., 2017b; Uematsu et al., 2017). In mice performing a 5-choice serial reaction time task (5CSRTT) to assess attention in animals, inhibiting LC neurons using Gi-coupled designer receptors exclusively activated by designer drugs (DREADDs) reduced attention performance by increasing errors and trial omissions. In contrast, inhibiting dopaminergic ventral tegmental area neurons only affected motivation and response speed (Fitzpatrick et al., 2019). Activation of LC-NE release in the BLA also increases anxiety-like behaviors (McCall et al., 2017a) suggesting LC-BLA pathway activation may perpetuate anxiety-like behavioral outcomes.

**Fig. 1:**
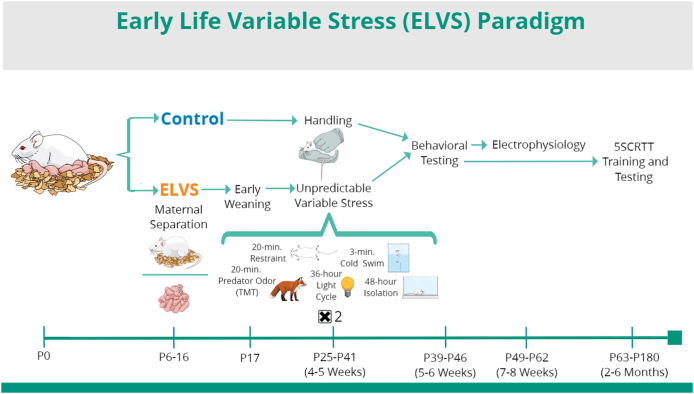
Early life variable stress (ELVS) paradigm and Experimental design.

Given the association of socioemotional stress, especially early in life, with the development of cognitive and affective disorders (Golm et al., 2020, 2021; Green et al., 2010; McLaughlin et al., 2019; Roy et al., 2016; Wade et al., 2022), we hypothesize that stress at critical times produces lasting changes in specific brain regions or circuits that shape cognitive and affective processing (e.g. LC-NE release in mPFC and amygdala). In parallel and based on sex-dependent variation in cognitive and affective disorder outcomes (Altemus et al., 2014; Gershon, 2002; Morken et al., 2023), we expect that these changes will also depend on biological sex. The application of valid pre-clinical animal models to identify mechanisms at play in these interactions is limited but needed to establish etiologies, prevention, and treatment for various cognitive and affective disorders. To address this outstanding issue of relevant animal models incorporating genetic determinants of cognitive/affective disruption susceptibility, we developed a model of early life stress-induced behavioral changes in BALB/cJ mice with an existing specific innate behavioral phenotype. The inbred strain of BALB/cJ mice expresses increased anxiety-like behaviors relative to C57BL/6J mice (Sartori et al., 2011), increased sensitivity to stress-induced behavioral alterations (Jacobson and Cryan, 2007; Tannenbaum and Anisman, 2003a, 2003b), altered central NE levels after foot shock (Shanks et al., 1994), and contain two polymorphisms within the gene encoding vesicular monoamine transporter 2 (VMAT2) (Crowley et al., 2005). As a relevant strain to assess the lasting effect of early life stress in animals with elevated basal anxiety-like behavior, we assessed the effect of a novel early life variable stress (ELVS) exposure paradigm (Fig. 1) on the behavior of male and female BALB/cJ mice. In parallel, we predicted that behaviors associated with LC function (e.g. anxiety and attention) would be impacted. To identify potential LC-NE mechanistic changes that may contribute to broad behavioral change, we used electrophysiology to further assess the synaptic activity and excitability of putative LC neurons in control and ELVS-exposed mice following behavioral testing.

## 2 Materials and methods

### 2.1 Animals

Male and female BALB/cJ (Jackson Labs, strain # 000651) originally purchased from Jackson Laboratory were bred in-house. All mice were group housed under a reversed 12-h light-dark cycle (lights on from 21:00 to 9:00) with ad libitum access to food and water. All manipulations and tests were performed during the animals’ active dark phase (9:00 to 21:00) unless otherwise stated. All procedures involving animals were conducted in accordance with protocols approved by the Institutional Animal Care and Use Committee (#2022–100) of Southern Illinois University - School of Medicine.

#### 2.1.1 Early life variable stress (ELVS) animal model

Male and female BALB/cJ mice were subject to either a two-phase (postnatal and adolescent) early life variable stress (ELVS) paradigm (Fig. 1) or treated as controls with all mice in a litter assigned to the same group. The ELVS paradigm consisted of two phases of manipulations, the first from postnatal days 6–17 and the second from postnatal days 28–40. Mice in the ELVS group were isolated from dams for 4 h/day during postnatal days 6–10 (P6-10) and weaned early at postnatal day 17 (P17). Pups were supplemented with nutrient gel (Nutra-gel from Bio-serve) from P17 to P21. From P28-42, ELVS mice were subjected to ten consecutive days of stressors with one stress exposure per day, the order of which varied randomly between cohorts/litters. These stressors included 20 min of tape restraint (2x), 20 min of 2,3,5-Trimethyl-3-thiazoline synthetic predator odor exposure (2x), 3 min of forced ice-cold swim in a 40 cm × 10 cm filled container (2x), 36 h of light cycle disruption via continuous light exposure (1x), and 48 h of social isolation (1x). Control male and female BALB/cJ mice were not separated from dams before weaning, weaned at P21, and were briefly handled 5 times per week for 10–15 min beginning at P28.

### 2.2 Innate behavioral tests

Behavioral tests were performed in low light conditions (15–20 lux red light). Mice were habituated to the testing room for 30 min prior to performing any assays, which were recorded digitally with a system for video tracking. Each apparatus was cleaned thoroughly with scent-free disinfectant between animals. All behavioral data were automatically analyzed by software (EthoVision XT 17.5, Noldus), except freezing behavior. Freezing was manually scored using EthoVision by an experimenter blinded to the group of the animal.

#### 2.2.1 Open field

Mice were placed in a white Plexiglas chamber (40 × 40 cm square open top chamber that is 30 cm high) for 30 min with total distance, time in the center (20 × 20 cm region equidistant from the box edges), center entrances, and freezing time calculated based on the center body point of the mouse.

#### 2.2.2 Elevated zero maze

Mice were placed in the open arm of an elevated circular platform 5 cm wide and 60 cm off the ground, with 2 parallel walled sections and 2 open sections in between, having an inner diameter of 40 cm. The amount of time spent (mouse center body point) in the open versus closed/walled arms/segments along with open arm entrances were evaluated. Mice entering/spending less time in the open arm were considered to have higher levels of anxiety-like behavior.

### 2.3 Operant five-choice serial reaction time task (5CSRTT)

For 5CSRTT testing of attention capacity, mice were trained and evaluated per standard protocols described here briefly (Fitzpatrick et al., 2018a, 2018b). Two weeks prior to experimentation, mice were water-restricted starting at 4 h of ad libitum access per day which was reduced to 2 h per day on testing days and ad libitum access on days when testing was not performed. During training sessions, mice were given sweetened condensed milk (800 ms pump time, 20 μL) as a reward for each trial when correctly performing the task and weighed to make sure they were at a proper weight after water restriction. The operant conditioning chamber (30.5 x 24.1 × 29.2 cm) consisting of 2 Plexiglas sidewalls, an aluminum front wall and back wall, and a stainless-steel grid floor was used as the 5CSRTT apparatus (Lafayette Instruments). The front wall contains five nose poke apertures (2.5 x 2.2 × 2.2 cm each) with each aperture containing a light-emitting diode (LED) and an infrared sensor cable to detect mouse nose insertions. The mice were trained (progressive shorting of intertrial interval and stimulus duration) for 30 min daily 5–7 days a week to reach baseline performance with an intertrial interval (ITI) of 5 s and the stimulus duration of 0.8 s. To pass baseline testing, mice were required to have an accuracy above 80% and omissions below 30% for three consecutive sessions. After reaching these threshold performance criteria, they were assessed again on three different test variations with either shorter ITI, longer ITI, or shorter stimulus duration. Response accuracy (% correct) and omitted trials (% omission) for each variable condition were calculated as the primary performance variables.

### 2.4 Ex vivo acute brian slice preparation

Animals were anesthetized with 4% isoflurane, followed by intracardial perfusion with ice-cold oxygenated N-methyl-d-glucamine (NMDG) artificial cerebrospinal fluid (NMDG-ACSF), which contained (in mM): 92 NMDG, 2.5 KCl, 0.5 CaCl2, 10 MgCl2, 1.2 NaH2PO4, 30 NaHCO3, 20 HEPES, 25 d-glucose, 2 ascorbic acid, 2 thiourea, and 3 sodium pyruvate and had an osmolarity of 300–310 mOsm with pH adjusted to 7.3–7.4 with HCl. Coronal slices (250 μm) that included the LC were acquired in ice-cold NMDG-ACSF with a Compresstome vibrating microtome (Processionary Instruments, LLC) and were transferred to a holding chamber containing NMDG-ACSF at 35 °C where the NaCl concentration increased steadily over 25 min. After 30 min, slices were transferred to a modified HEPES-based ACSF (HEPES-ACSF, 35 °C), which contained (in mM): 92 NaCl, 2.5 KCl, 2 CaCl2, 2 MgCl2, 1.2 NaH2PO4, 30 NaHCO3, 20 HEPES, 25 d-glucose, 2 ascorbic acid, 2 thiourea, 3 sodium pyruvate, and 3 myo-inositol and had an osmolarity of 300–310 mOsm with pH 7.3–7.4. After incubating slices in HEPES-ACSF at 35 °C for 1 h, slices were maintained in HEPES-ACSF at room temperature until being transferred to the recording chamber where they were continuously perfused at a 3–5 ml/min with oxygenated ACSF, which contained (in mM): 125 NaCl, 2.5 KCl, 2 CaCl2, 2 MgCl2, 1NaH2PO4, 26 NaHCO3, 20 D-glucose, 2 ascorbic acid, and 3 myo-inositol and had an osmolarity of 310–320 mOsm with pH 7.3–7.4.

### 2.5 Ex vivo acute brain slice whole-cell electrophysiology

Neurons in the LC were visualized using an upright microscope (Olympus BX51WI) with a 40X water immersive objective. Whole-cell patch-clamp recordings were performed at 32–34 °C maintained with an in-line solution heater from neurons within the LC visually identified (infrared differential interference contrast) based on regional landmarks and relationship to the 4th ventricle. Using a horizontal puller (P1000, Sutter Instruments), patch pipettes were pulled from filamented borosilicate glass capillaries (outer diameter 1.5 mm, inner diameter 0.86, Sutter Instruments), having a tip resistance of 3–5 MΩ when filled with potassium gluconate-based internal solution that contained (in mM): 139.6 mM K-gluconate, 0.4 mM KCl, 4 mM NaCl, 0.5 mM CaCl_2_, 10 mM Hepes, 5 mM EGTA free acid, 4 mM ATP Mg salt, 0.5 mM GTP Na salt with an osmolality 285–290 mOsm and the pH adjusted to 7.2–7.3 with KOH. For voltage-clamp recordings, 5 mM QX-314 iso-osmotically replaced NaCl to block voltage-gated Na + channels. All signals were acquired at 20 kHz and low-pass filtered at 10 kHz by a Digidata1440 digitizer and a MultiClamp 700B amplifier. Data were collected from the neurons with an input resistance >100 MΩ. If the series resistance for a given recording was >35 MΩ or changed by more than 20% throughout a recording, the data were rejected for analysis. Liquid junction potentials remained uncorrected in all cases.

### 2.6 Quantification and statistical analysis

Mice of both sexes from control and stressed (ELVS) groups were evaluated for behavioral and electrophysiology experiments. All results are expressed as the mean ± SEM and alpha levels of *p* < 0.05 were considered significant. Two-way ANOVAs (sex, stress) or repeated measures ANOVAs (5CSRTT test or current injection steps, sex, stress) were used to identify significant main effects or interactions of stress and sex. Since follow-up pairwise comparisons were only made to assess stress effects and multiple comparisons were not made, the likelihood of family-wise or Type I error was low. Therefore, based on ANOVA results, t-tests were used to evaluate pairwise comparisons that were made between control and ELVS groups within each sex or with data from both sexes combined. When data variance was not homogeneous, a Welch's *t*-test was used for pairwise comparisons, a non-parametric Kruskal Wallis H test with a Dunn's post-hoc test was performed in place of two-way ANOVAs, or the alpha level threshold was increased to *p* < 0.01 for repeated measures ANOVAs. Statistical test results are not provided for ANOVA main effects or interactions or pairwise comparisons (t-tests) when *p* > 0.1, except where relevant. SPSS 29 (IBM) and Igor Pro 8 (Wavemetrics) were used for automated and manual determination of dependent variable values in EthoVision XT 17.5. Data analyses and graphing were conducted in Clampfit 10.0, Easy Electrophysiology, Igor Pro 8, and SPSS 29.0 (IBM).

## 3 Results

### 3.1 ELVS exposure leads to hyperactivity and female-specific increased anxiety in adolescence

**Fig. 2:**
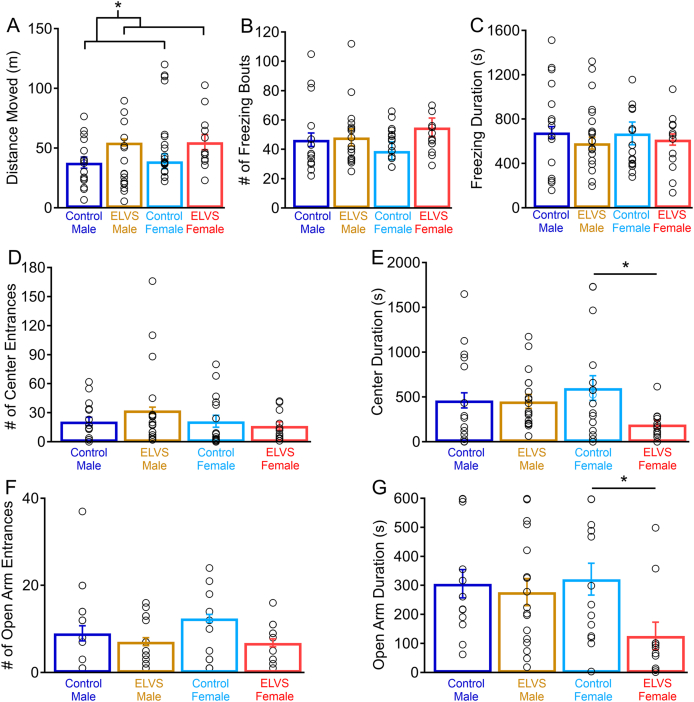
**ELVS mice are hyperactive while female ELVS mice have increased anxiety-like behaviors.** (**A-E**) Mean ± SEM (bars) and individual (circles) open field (OF) measures for total distance moved in meters (m) (stress: *F*(1,61) = 5.89, *p* = 0.018 *t*(61) = −2.50, *p* = 0.015) (**A**), number of freezing (**B**), the freezing time (**C**), OF center area entrances (**D**), and OF center area time (*H*(3, *n* = 62) = 7.61, *p* = 0.055; males: *p* = 0.284; females: *p* = 0.015; **E**). (**F** & **G**) Mean ± SEM (bars) and individual (circles) values in the EZM for the number of times mice entered the open arm (stress: *F*(1,53) = 3.57, *p* = 0.065; **F**) and the cumulative time spent in the open arm (stress: *F*(1,53) = 4.47, *p* = 0.039, *t*(52) = 1.81, *p* = 0.077; sex-stress interaction: *F*(1,53) = 2.49, *p* = 0.12; **G**) identifying a significant difference in open arm time only in females (males: *t*(27) = 0.39, *p* = 0.70; females: *t*(23) = 2.64, *p* = 0.015). OF N = 12–19 mice/group, EZM N = 11–17 mice/group, ∗*p* < 0.05 with two-tailed t-tests and two-way ANOVA.

To determine how early life stress impacts BALB/cJ mice which display an elevated basal level of anxiety-like behavior, we subjected male and female BALB/cJ mice to an early life variable stress (ELVS) paradigm described above (Fig. 1). At 6 weeks of age, control and ELVS male and female mice were evaluated on open field (OF) and elevated zero maze (EZM) behavioral assays to assess locomotive and anxiety-like behaviors. The total distance moved in the OF was increased in ELVS-exposed mice regardless of sex. ELVS (male and female) mice moved more than control mice during the 30 min of OF testing (Fig. 2A), suggesting elevated basal activity and exploratory behavior. Although freezing behavior or the number of entries into the center of the OF (Fig. 2B–D) were not different between groups, there was a strong trend (*p* = 0.055) toward group differences in the amount of time spent in the center of the OF. This trend was driven by a significant reduction in the OF center time for ELVS female mice relative to controls, but not males (Fig. 2E). In an additional assessment of anxiety, the EZM assay, neither ELVS nor sex affected entries into open arms (Fig. 2F). However, ELVS exposure significantly affected EZM open arm time in female ELVS mice (Fig. 2G). Given the sex-specific effect of the anxiety-like behavior measure in the open field and our hypothesis regarding sex-specific changes in behavior due to stress, additional comparisons of stress effects indicated a significant reduction in EZM open arm time (increased anxiety-like behavior) in females, but not males (Fig. 2F and G). Together, behavior in OF and EZM indicate that ELVS leads to overall hyperactivity in BALB/cJ mice regardless of sex, but only increases basal anxiety-like behavior in females.

### 3.2 ELVS exposure impairs attention capacity in adulthood

**Fig. 3:**
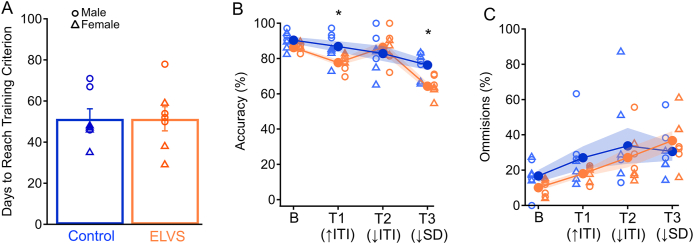
**ELVS results in impaired attention in adulthood. (A)** Mean ± SEM (bars) and individual (circles) number of five-choice serial reaction time task (5CSRTT) training days to reach training criterion (sex: *F*(1,13) = 6.722, *p* = 0.027, *t*(12) = 2.79, *p* = 0.016). (**B** & **C**) Mean ± SEM (filled circles with shaded region) and individual (open circles = males, open triangles = females) accuracy (test sequence and stress: *F* (1,13) = 3.74, *p* = 0.022); T1, stress: *t*(12) = 2.60, *p* = 0.023; T3, stress: *t*(12) = 3.43, *p* = 0.005; **B**) and omission percentages (test sequence, stress and sex interaction: *F* (1,13) = 6.49, *p* = 0.002; males T2: t(5) = 4.78, p = 0.005; **C**) on 5CSRTT baseline (B), test 1 (T1) with increased intertrial interval values (↑ITI), test 2 (T2) with reduced intertrial interval values (↓ITI), and test 3 (T3) with reduced stimulus duration values (↓SD). N = 7 per group. ∗*p* < 0.05 with pairwise two-tailed t-tests and repeated measures three-way ANOVA.

Upon completion of innate behavioral assays, we initiated water restriction and training of a subset of BALB/cJ control and ELVS mice to perform the five-choice serial reaction time task (5CSRTT) as an assessment of attention capacity. Stress exposure did not impact the time taken to reach the accuracy and omission threshold criteria during the training period (Fig. 3A), but males (59.7 ± 4.6 days) took significantly longer than females (43.1 ± 3.8 days) to reach the learning performance criteria as a whole (Fig. 3A). Measures of baseline performance were taken from the third successful completion of the final training phase (5-s ITI and 0.8-s SD; Fig. 3B and C), followed by daily testing with three different variable changes with either increased ITI (test 1), decreased ITI (test 2), or shortened stimulus duration (test 3). Although there were no significant accuracy changes due to interactions of the testing sequence with sex or sex and ELVS exposure together, there was a significant interaction of ELVS exposure alone with the test sequence. Specifically, ELVS exposure led to reduced trial accuracy with increased intertrial interval or decreased stimulus duration (Fig. 3B). These reductions in performance accuracy on attention tasks that require prolonged maintenance of attention to stimuli (increased ITI and shortened stimulus duration) suggest that vigilance may be perturbed due to ELVS exposure in BALB/cJ mice generally, in addition to hyperactivity.

### 3.3 ELVS alters spontaneous postsynaptic inhibitory currents in LC neurons of male mice

**Fig. 4:**
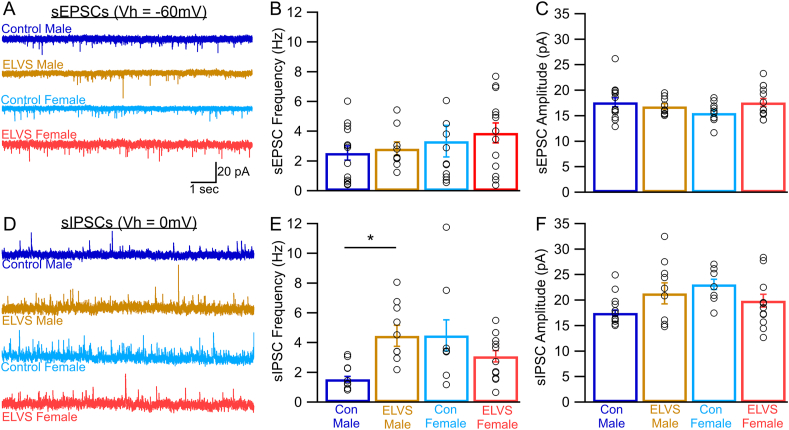
**ELVS selectively increases inhibitory synaptic tone in male LC neurons.** (**A, D**) Representative voltage clamp recording traces of control and ELVS putative LC neuron spontaneous excitatory postsynaptic currents (sEPSCs, Vhold = −60 mV, **A**) and inhibitory postsynaptic currents (sIPSCs, Vhold = 0 mV, **D**). (**B, C**) Mean ± SEM (bars) and individual (circles) sEPSC frequency (**B**) and amplitude (**C**). (**E, F**) Mean ± SEM (bars) and individual (circles) sIPSC frequency (**E**) and amplitude (**F**) indicate sIPSC frequency (sex-stress: *F* (1,35) = 8.25, *p* = 0.007) is selectively elevated in male ELVS (*t*(16) = 3.80, *p* = 0.002) LC neurons with sIPSC amplitude (sex-stress interaction: *F* (1,35) = 5.34, *p* = 0.027) not different between groups (*t*-test *p* > 0.05). N = 3 mice per group, sEPSC: n = 9–13 cells/group, sIPSC: n = 8–10 cells/group, ∗*p* < 0.05 with pairwise two-tailed t-tests and two-way ANOVA.

Given the functional reciprocal connectivity of the LC with the amygdala and PFC to shape both anxiety-like and attention behaviors, we hypothesized that changes in the central adrenergic system may underpin these altered behaviors. First, to assess spontaneous excitatory and inhibitory synaptic activity within LC neurons, we performed voltage-clamp electrophysiology in putative LC neurons from acute brain slices *ex vivo*. To isolate spontaneous excitatory postsynaptic currents (sEPSCs), neurons were voltage-clamped at −60 mV which was equal to the E_Cl_ (Fig. 4A). We did not observe any significant differences in sEPSC amplitude or frequency (Fig. 4B and C). To next isolate spontaneous inhibitory postsynaptic currents (sIPSCs), putative LC neurons were voltage-clamped at 0 mV which was near the cation reversal potential (Fig. 4D). Evaluation of sIPSCs identified a significant increase in sIPSC frequency in ELVS male mice relative to male controls without stress-related changes in sIPSC activity in females (Fig. 4E and F).

### 3.4 ELVS LC neurons display reduced evoked and spontaneous excitability

**Fig. 5:**
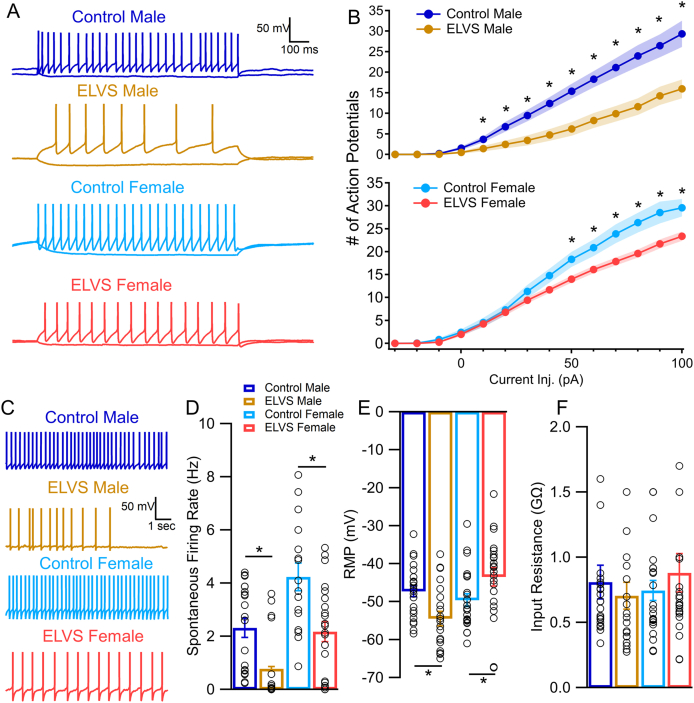
**ELVS results in reduced LC neuron excitability.** (**A**) Representative traces of current clamp recordings from control and ELVS putative LC neurons in response to +80/-30 pA current injection (1 s) from each neuron's natural resting membrane potential. (**B**) Mean ± SEM (filled circles with shaded region) action potential (AP) number in response to current (pA) injection at each level for males (upper) and females (lower) of each group (sex: *F*(1,83) = 8.41, *p* = 0.005**;** stress: *F*(1,83) = 19.22, *p* < 0.001). (**C**) Representative traces of spontaneous firing (10 s) recordings from putative LC neurons from each group. (**D-F**) Mean ± SEM (bars) and individual (circles) spontaneous firing rates (sex: *F*(1,72) = 14.50, *p* < 0.001; stress: *F*(1,72) = 17.29, *p* < 0.001; males: *t*(33) = 2.62, *p* = 0.013; females: *t*(36) = 3.29, *p* = 0.002; **D**), resting membrane potential (sex: *F*(1,86) = 4.65, *p* = 0.034; sex-stress interaction: *F*(1,86) = 1170, *p* = 0.001; males: *t*(41) = 2.82, *p* = 0.007; females: *t*(42) = −2.14, *p* = 0.038; **E**), and input/membrane resistance (**F**). n = 14–24 cells/group from N = 3 mice/group. ∗*p* < 0.05 with two-tailed t-tests and three-way repeated measures ANOVA (**B**) or two-way ANOVA (**D-F**).

We next evaluated LC neuron excitability and additional membrane properties. ELVS mice displayed reduced current injection-evoked excitability in both sexes (Fig. 5A and B). Both stress exposure and sex affected spontaneous firing rate of LC neurons, which was reduced in both male and female ELVS mice relative to control mice (Fig. 5C and D). We also observed sex-specific changes in the resting membrane potential, with ELVS leading to membrane potential hyperpolarization in LC neurons from male and depolarization in LC neurons from female mice (Fig. 5E). Without significant changes in the input/membrane resistance (Fig. 5F) of LC neurons, this resting potential change in male ELVS mice relative to controls accounts for evoked and spontaneous firing rate changes observed in this group. However, these changes alone, without meaningful changes in synaptic drive in LC neurons from female mice exposed to ELVS failed to explain the reduced excitability observed in this group.

### 3.5 ELVS LC neurons show a sex-specific increase in action potential delay time

**Fig. 6:**
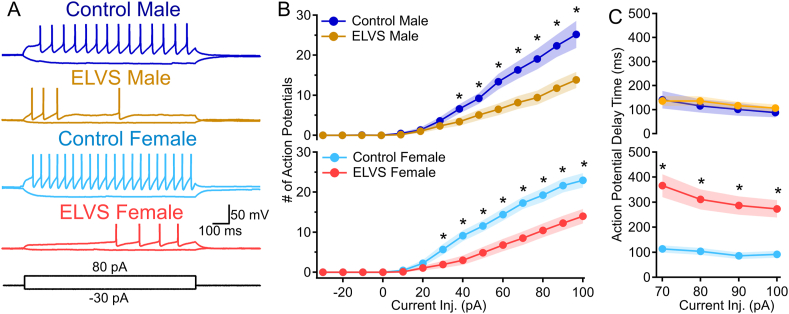
**ELVS results in a female-specific increase in LC neuron action potential delay time.** (**A**) Representative traces of current clamp recordings from control and ELVS putative LC neurons in response to +80/-30 pA current injection (1 s) with the initial membrane potential adjusted to −60 mV with current injection. (**B-C**) Mean ± SEM action potential (AP) number (stress: *F*(1,74) = 18.49, *p* < 0.001) (**B**) and first action potential delay time (**C**) due to 70–100 pA current injection with a starting membrane potential of −60 mV (sex-stress interaction: *F*(1,69) = 13.90, *p* < 0.001). n = 14–23 cells/group, N = 3 mice/group, ∗*p* < 0.05 with two-tailed t-tests and three-way repeated measures ANOVA.

Since resting potential changes sex-dependently with ELVS, likely due to increased inhibitory drive (Fig. 4), only explained excitability reductions in LC neurons of male mice, we next assessed LC neuron evoked excitability when the resting membrane potential was adjusted with constant current injection to −60 mV (∼E_Cl_, to nullify the effects of inhibitory drive; Fig. 6A). With an initial resting potential of −60 mV, there remained a significant effect of ELVS exposure on current injection-evoked firing across the range of current values in LC neurons from both sexes (Fig. 6B), but the interaction between sex and ELVS exposure observed at natural resting potentials was lost. However, for consistency, the data are shown with each sex separately. Stress effects in males are comparable at the natural resting potential and at −60 mV, but this configuration identifies greater changes in excitability due to ELVS exposure in females (Fig. 6B). Unlike at more depolarized natural resting potentials, putative LC neurons specifically from ELVS female mice displayed a delay before the first action potential across nearly all current injections (Fig. 6A). A comparison of first action potential delay times across groups for all neurons eliciting action potentials in response to 70–100 pA injection identified a sex and ELVS exposure interaction affecting the delay time (Fig. 6C). The first action potential delay was significantly different between control and ELVS in LC neurons only from female mice in response to each current injection, but not males (Fig. 6C).

## 4 Discussion

### 4.1 Summary of findings

To develop an animal model to understand the underlying mechanisms involved in early life stress-induced changes in affective and cognitive behaviors, we found that combining maternal deprivation in early life and unpredictable stress in adolescence resulted in behavioral changes. Specifically, motor hyperactivity occurred after stress in male and female BALB/cJ along with female-specific increased anxiety-like behavior in adolescence (Fig. 2). We also observed impaired attention performance in the 5CSRTT, suggestive of reduced vigilance in both sexes persisting into early adulthood (Fig. 3). Paralleling these behavioral deficits, LC neuron excitability was reduced in mice of both sexes after early life stress through different mechanisms (Fig. 5). Relative to LC neurons from control mice, the reduced excitability of LC neurons of male mice exposed to ELVS appeared due to hyperpolarized resting membrane potentials (Fig. 5), possibly a result of increased inhibitory synaptic activity (Fig. 4). On the other hand, when accounting for resting potential, only LC neurons in female ELVS mice displayed a dramatic increase in action potential delay time in response to depolarizing current (Fig. 6), possibly due to differential activation of voltage-gated ion channels. These findings suggest that early life stress impacts LC physiology in sex-specific ways that may contribute to persistent attention deficits and/or anxiety-like behavior.

### 4. 2 The LC as an interface between stress and affective/cognitive behaviors

In considering a mechanism for ELVS-induced changes in LC neuron activity, release and sex-specific signaling of corticotropin releasing factor (CRF) in the LC may be a key factor. The LC is innervated by CRF-containing neuron terminals from the paraventricular hypothalamic nucleus (PVN), CeA, Barrington's nucleus, and the nucleus paragigantocellularis (Koegler-Muly et al., 1993; Valentino et al., 1992). Intracerebroventricular CRF injection into the brain and direct CRF application onto LC neurons have been shown to increase LC neuron spontaneous firing rates (Valentino et al., 1983) via activation of CRF_1_ receptors (Sánchez et al., 1999). CRF_1_ couples to G_i_, G_s_, and G_q_ pathways, with notable sex differences in preferred coupling and receptor internalization shown in the LC in rodent studies (Bangasser and Wiersielis, 2018; Curtis et al., 2006). It is perhaps these sex differences in CRF_1_ signaling in the LC that may lead to sex-specific persistent changes in basal LC physiology affecting inhibition and excitability identified in the data presented here. Both acute and chronic stress have been shown to alter LC metabolism and NE levels in its target brain regions (Fan et al., 2014; Keshavarzy et al., 2015). While multiple synaptic and signaling factors regulate LC neuron activity, levels of CRF act directly on LC neuron CRF_1_ to increase LC neuron firing and dose-dependently alter performance in attention set shifting (Cole et al., 2016; Curtis et al., 2006; Snyder et al., 2012). It has been shown that CRF positive inputs from the amygdala increase LC tonic firing to increase anxiety-like and aversive behaviors (McCall et al., 2015). These studies reflect the impact of stress via CRF signaling within the LC on cognitive and affective behaviors.

The LC is involved in both anxiety and attention, primarily due to its modulation of the amygdala and mPFC (Aston-Jones et al., 1999; Borodovitsyna et al., 2020; Campese et al., 2017; Lim et al., 2010; Mair et al., 2005; McCall et al., 2017a; Van Stegeren et al., 2005; Zhang et al., 2023). In male Sprague Dawley rats immediately after acute stress, levels of cfos (i.e. a genetic marker for neuronal activity) were increased in LC neurons that projected to the CeA and mPFC in parallel to increased anxiety-like behavior. In this same study, subsequently identified electrophysiological changes in these LC neurons were also observed, but stress-induced excitability changes differed between CeA- and mPFC-projecting LC neurons. DREADD-dependent reversal of a stress-induced increase in excitability of LC neurons projecting to CeA caused a reduction in stress-induced anxiety-like behavior (Borodovitsyna et al., 2020). Comparing this work determining how stress-induced increases in CeA-projecting LC neuron excitability shapes anxiety-like behavior to the data presented here where we find lasting reductions in LC neuron activity after stress highlights two important considerations. First, acute effects of stress likely produce one set of physiological changes while lasting effects of stress produce another, whether via synaptic, ensemble, or intracellular mechanisms. Second, our work does not make a distinction between LC neuron types based on projection which may identify subpopulations that indeed generate lasting responses to stress that differ between projection types as observed in response to acute stress.

LC neurons projecting to the mPFC have also been shown to regulate attention performance (Bouret and Sara, 2004; Hallock et al., 2024). LC projections to the mPFC prelimbic area were shown to be recruited during the rodent continuous performance test (rCPT), with LC theta frequencies leading during correct responses and prelimbic mPFC neurons leading in gamma frequencies during incorrect responses (Hallock et al., 2024). In humans, acute and chronic stress has been shown to impact attention performance (Liu et al., 2020; Rojas-Thomas et al., 2023). Acute psychosocial stress-induced impairments also correlated with reduced pupil dilation during attention tasks, which indicates reduced phasic NE release (Rojas-Thomas et al., 2023). All together, these studies show how stress impacts LC function, and its potential role in modulating anxiety and attention behaviors, both of which are affected in cognitive and affective processing disorders (Jensen and Steinhausen, 2015; Martínez et al., 2016; Saccaro et al., 2021; Shaw et al., 2014; Willcutt et al., 2005), As a corollary, the LC physiology changes that occur after ELVS provide insight into a mechanism by which early and transient stress induces lasting disruption of LC-NE function to drive behavioral change.

### 4.3 Impacts of reduced LC neuron excitability on LC network-related processing

The additional female-specific increase in the action potential delay time when cells were held at a −60 mV further indicates that there may be stress-sensitive mechanistic changes that would alter excitability with or without changes in input (Jerng et al., 2004). Rather, the female-specific changes in LC neurons of ELVS mice would largely affect how excitatory input is integrated to modulate LC neuron firing over time. Since it has been shown that LC neurons must transition from different firing modes (i.e. tonic vs. phasic) in order to shift into different optimal attention states depending on the task (Howells et al., 2012), changes in how these transitions occur may rely on mechanisms that shape both spontaneous and evoked excitability. An overall reduction in LC neuron excitability would impact both stimulated and spontaneous LC synchronous activation involved in determining cortical states that are associated with behavioral state transitions (Carter et al., 2010; Hayat et al., 2020; Marzo et al., 2014; Noei et al., 2022). Aside from the highly synchronized en masse LC population stimulation leading to an overall activated cortical state, there are alternatively diverse cortical states that are activated by different LC ensembles or groups of LC neurons (Noei et al., 2022). Rendering LC neurons less spontaneously active and responsive to input may reduce the diversity of cortical states and parallel behavioral flexibility. In the data we show here, attention deficits in ELVS mice when the intertrial interval is elongated and the stimulus duration is shortened, both increasing attention demand (increasing focused attention time or vigilance). Reduced LC neuron excitability could impair the transition into cortical states required for these specific attention-demanding modes. Reduced LC neuron excitability may also impact anxiety behaviors, with reduced mPFC-projecting LC neuron excitability found in mice with increased anxiety-like behavior after acute stress (Borodovitsyna et al., 2020). Together, these data suggest that the hypoexcitable LC neurons projecting to the mPFC may negatively impact both anxiety and attention behaviors.

### 4.4 Mouse strain and responses to early life stress

Rather than using a more common laboratory mouse strain like the C57BL/6J strain, we deliberately chose BALB/cJ mice based on their unique behavioral phenotype prone to elevated anxiety-like behavior. While the freezing behavior of this strain of mice brings some challenges to data analysis (see Section 4.5 below), understanding how a predisposition for elevated basal anxiety-like behavior interacts with stress exposure early in life is key in identifying potential mechanisms that lead to variable long-lasting behavioral change. Due to innate behavioral phenotypic differences, we hypothesize that BALB/cJ mice would have contrasting behavioral outcomes to C57BL/6J after ELVS exposure. Since C57BL/6J mice have reported differences in basal and stress-induced anxiety-like behaviors and monoamine metabolism, we predict that they may be more resistant to ELVS-induced behavioral and LC electrophysiological changes (Sartori et al., 2011; Shanks et al., 1994; Tannenbaum and Anisman, 2003a, 2003b). The studies assessing strain differences in monoamine levels after acute stress were conducted in males only, therefore there may be sex differences in C57BL/6J mice that remain unknown (Shanks et al., 1994; Tannenbaum and Anisman, 2003a). In the future, we plan to assess ELVS effects on male and female C57BL/6J mouse behavior and LC electrophysiology to determine how they differ from BALB/cJ or other mouse strains and if there are sex differences within these measures. This will contribute to our understanding of how both genetic background and sex interact with early life stress as influential biological variables for psychiatric disorder risks. The LC may be a target region for behavioral alteration sensitivity due to early life stress, providing direction for treating sensitive clinical populations.

### 4.5 Pitfalls and limitations

The limitations of this study primarily stem from the 5CSRTT training timeline and using the BALB/cJ mouse strain that displays unique behavioral characteristics. 5CSRTT training takes several months, therefore it is difficult to test many animals due to time constraints. This makes assessing sex differences in these behaviors difficult, and there is a lack of studies including females in this task to which results may be compared. This task is taken from the clinical version of the task, rendering results more readily translatable to data from studies in humans. The BALB/cJ mice in general move less in novel environments when compared to C57BL/6J mice and exhibit more frequent and prolonged freezing behavior. This does complicate the interpretation of anxiety-like behaviors, especially when a mouse freezes in the center or open arm of the OF or EZM apparatuses respectively. In this study, we assessed the excitability of the LC neuron population as a whole as opposed to dividing the population based on where the neurons project to (i.e. CeA or mPFC). However, at least one study has determined that the nature or direction of LC neuron excitability changes after acute stress depends on which brain areas those LC neurons target (Borodovitsyna et al., 2020). With these data in mind, coupling the approach described here with viral tract tracing to identify neuronal population-specific changes may further clarify an interaction of molecular mechanisms with circuit involvement.

## 5 Conclusions and future directions

Exposure to stress, especially at critical periods in early life, has clinically relevant impacts on behavior neuropsychiatric disorder risk. In general, women have a greater risk for stress-related disorders. Women are twice as likely to have depression, GAD, and post-traumatic stress disorder (PTSD) when compared to men, with this difference emerging after adolescence (Kessler et al., 2004; Morken et al., 2023). Sex differences in adolescent stress effects on affective behaviors could explain the sex differences in anxiety-like behavioral outcomes seen in our model. Typically, boys more commonly suffer from neurodevelopmental disorders such as ADHD or autism that are also associated with ACEs (Bölte et al., 2023). In childhood, the male-to-female ratio of ADHD prevalence is 3:1, but approaches 1:1 in adulthood (Bölte et al., 2023; da Silva et al., 2020). In this study, 5CSRTT attention testing is performed during adulthood, suggesting that in both males and females, there are lasting attention deficits in our ELVS model. Further investigation regarding behavioral outcomes when isolating the early life and adolescent components of the ELVS paradigm will help to understand if each component is necessary for behavioral deficits, if a certain component is responsible for sex-specific effects, or if only one component is needed for the behavioral outcomes we have observed.

The results of this study support further exploration into LC-NE-related treatment targets for ACE-related disorders, particularly when attention deficits are present. It also suggests sex is a crucial biological variable involved in determining psychiatric outcomes due to ACE(s) exposure and treatment. This study suggests early life stress results in reduced LC neuron excitability in both sexes, but the causal mechanism and functional impacts of this change may differ between males and females resulting in sex differences in behavioral outcomes. Further investigation of whether similar treatment targets will work in both sexes or if more specific targets depending on sex may be more beneficial at ameliorating affective and/or cognitive behavioral deficits.

## Ethics approval

All procedures involving animals were performed in accordance with protocols approved by the Institutional Animal Care and Use Committee at Southern Illinois University – School of Medicine.

## Availability of data and materials

The datasets used and/or analyzed during the current study are available from the corresponding author upon reasonable request.

## Funding

This work was supported by a Research Seed Grant and laboratory operating funds provided to BDR by 10.13039/100008570Southern Illinois University – School of Medicine

## CRediT authorship contribution statement

**Savannah Brannan:** Writing – review & editing, Writing – original draft, Visualization, Validation, Methodology, Investigation, Formal analysis, Data curation, Conceptualization. **Lauren Garbe:** Formal analysis. **Ben D. Richardson:** Writing – review & editing, Visualization, Validation, Supervision, Project administration, Methodology, Funding acquisition, Data curation, Conceptualization.

## Declaration of competing interest

The authors declare that they have no known competing financial interests or personal relationships that could have appeared to influence the work reported in this paper.

## Data Availability

Data will be made available on request.

## ABBREVIATIONS

(ACEs): Adverse Childhood Experiences
(ADHD): Attention deficit hyperactivity disorder
(GAD): Generalized anxiety disorder
(NE): Norepinephrine
(LC): Locus coeruleus
(CRF): Corticotropin-releasing hormone/factor
(ELVS): Early life variable stress
(VMAT2): Vesicular monoamine transporter 2
(mPFC): Medial Prefrontal cortex
(5CSRTT): 5-choice serial reaction time task
(DREADDs): Designer receptors exclusively activated by designer drugs
(CeA): Central amygdala
(p-ERK): Phosphorylated extracellular signal-regulated kinase
(CRF_1_): CRF receptor 1
(OF): Open field
(EZM): Elevated zero maze
(sEPSCs): Spontaneous excitatory postsynaptic currents
(sIPSCs): Spontaneous inhibitory postsynaptic currents
(PVN): Paraventricular hypothalamic nucleus
(PTSD): Post-traumatic stress disorder
